# Supervised Machine Learning Applied to Automate Flash and Prolonged Capillary Refill Detection by Pulse Oximetry

**DOI:** 10.3389/fphys.2020.564589

**Published:** 2020-10-06

**Authors:** Ryan Brandon Hunter, Shen Jiang, Akira Nishisaki, Amanda J. Nickel, Natalie Napolitano, Koichiro Shinozaki, Timmy Li, Kota Saeki, Lance B. Becker, Vinay M. Nadkarni, Aaron J. Masino

**Affiliations:** ^1^Department of Anesthesiology and Critical Care Medicine, Children’s Hospital of Philadelphia, Philadelphia, PA, United States; ^2^Nihon Kohden Innovation Center, Cambridge, MA, United States; ^3^Department of Respiratory Therapy, Children’s Hospital of Philadelphia, Philadelphia, PA, United States; ^4^Department of Emergency Medicine, Donald and Barbara Zucker School of Medicine at Hofstra/Northwell, Hempstead, NY, United States

**Keywords:** perfusion, oximetry, supervised machine learning, intensive care units, pediatrics, gradient boosting

## Abstract

**Objective:**

Develop an automated approach to detect flash (<1.0 s) or prolonged (>2.0 s) capillary refill time (CRT) that correlates with clinician judgment by applying several supervised machine learning (ML) techniques to pulse oximeter plethysmography data.

**Materials and Methods:**

Data was collected in the Pediatric Intensive Care Unit (ICU), Cardiac ICU, Progressive Care Unit, and Operating Suites in a large academic children’s hospital. Ninety-nine children and 30 adults were enrolled in testing and validation cohorts, respectively. Patients had 5 paired CRT measurements by a modified pulse oximeter device and a clinician, generating 485 waveform pairs for model training. Supervised ML models using gradient boosting (XGBoost), logistic regression (LR), and support vector machines (SVMs) were developed to detect flash (<1 s) or prolonged CRT (≥2 s) using clinician CRT assessment as the reference standard. Models were compared using Area Under the Receiver Operating Curve (AUC) and precision-recall curve (positive predictive value vs. sensitivity) analysis. The best performing model was externally validated with 90 measurement pairs from adult patients. Feature importance analysis was performed to identify key waveform characteristics.

**Results:**

For flash CRT, XGBoost had a greater mean AUC (0.79, 95% CI 0.75–0.83) than logistic regression (0.77, 0.71–0.82) and SVM (0.72, 0.67–0.76) models. For prolonged CRT, XGBoost had a greater mean AUC (0.77, 0.72–0.82) than logistic regression (0.73, 0.68–0.78) and SVM (0.75, 0.70–0.79) models. Pairwise testing showed statistically significant improved performance comparing XGBoost and SVM; all other pairwise model comparisons did not reach statistical significance. XGBoost showed good external validation with AUC of 0.88. Feature importance analysis of XGBoost identified distinct key waveform characteristics for flash and prolonged CRT, respectively.

**Conclusion:**

Novel application of supervised ML to pulse oximeter waveforms yielded multiple effective models to identify flash and prolonged CRT, using clinician judgment as the reference standard.

**Tweet:**

Supervised machine learning applied to pulse oximeter waveform features predicts flash or prolonged capillary refill.

## Introduction

Shock is a medical emergency associated with high morbidity and mortality in both children and adults. Early identification of shock is critical to appropriately intervene and improve outcomes (Rivers and Ahrens, 2008). Physical examination and assessment of perfusion is a critical component of shock evaluation and plays a key role in determining immediate management before invasive laboratory and hemodynamic measures can be obtained (Cecconi et al., 2014; American Heart Association, 2016). Guidelines including the Pediatric Advanced Life Support and the American College of Critical Care Medicine guidelines for pediatric and neonatal septic shock recommend capillary refill time (CRT) measurement as a component of early shock assessment (Fleming et al., 2015a; Davis et al., 2017). These groups recognize both warm (vasodilated) and cold (vasoconstricted) states as possible clinical presentations of shock. Flash (very fast) and more commonly prolonged CRT have been studied as an indicator of hemodynamic status and predictor of critical illness in patients with shock (Van den Bruel et al., 2010; Fleming et al., 2015a, 2016).

CRT assessment has wide variability in reported inter-rater and intra-rater reliability (Pickard et al., 2011; Fleming et al., 2015b; Shinozaki et al., 2018). This variability may be explained by inconsistent measurement technique with regard to body site location, aspect of the digit observed, duration of pressure application, and amount of pressure applied (Pandey and John, 2013; Fleming et al., 2015b). To improve reproducibility in CRT measurement, an automated CRT device with a modified pulse oximeter was created. This device is placed on the patient’s index or middle finger and measures the change in red and infrared light absorption when manual pressure is applied and subsequently released to estimate CRT (referred to here as Capillary Refill index, CRi) (Morimura et al., 2015). Preliminary data showed reasonable correlation between device measured CRi with blood lactate levels and clinician-assessed CRT (Morimura et al., 2015; Oi et al., 2018; Shinozaki et al., 2019a).

Machine learning analysis has been recently applied to various modalities in shock assessment, including traditional hemodynamic and biochemical features, near infrared spectroscopy, and thermal images (Convertino et al., 2011; Liu et al., 2019; Nagori et al., 2019). Machine learning has also been applied to pulse oximeter waveforms for the detection of obstructive sleep apnea, oxygenation changes following ventilator adjustment, detection of blood pressure, and detection of blood glucose values (Monte-Moreno, 2011; Andrés-Blanco et al., 2017; Hornero et al., 2017; Ghazal et al., 2019; Mousavi et al., 2019). Machine learning analysis of pulse oximeter waveform data to detect shock state has been limited. The goal of this study was to apply machine learning techniques to pulse oximeter waveforms to develop the models for detection of the presence of flash and prolonged CRT determined by clinicians. We then externally validated the best performing model, and explored the physiologic significance of waveform features.

## Materials and Methods

### Definition of Normal, Flash, and Prolonged CRT

For our study, a sample was considered flash, normal, or prolonged if, based on clinician assessment: CRT <1.0 s, 1.0 ≤CRT <2.0 s, or CRT >2.0 s, respectively. In clinical practice, normal CRT is defined clinically as <2.0 s. Prolonged CRT is commonly defined as >2.0 or >3.0 s. Two to three seconds is considered potentially normal or indeterminate (Fleming et al., 2015a; American Heart Association, 2016; Davis et al., 2017). We chose CRT >2.0 s as a cutoff due to a lack of sufficient sample pairs with clinician-judged CRT >3.0 s for model training (82 pairs with CRT >2.0 s compared to only 19 pairs with CRT >3.0 s). Flash CRT, representative of an arterial vasodilatory state seen in patients with warm shock in the presence of warm extremities, bounding pulses, and widened pulse pressure (Davis et al., 2017), was defined as <1.0 s in this study.

### Study Subjects and Data Collection

This study of a secondary analysis of an existing dataset was approved by our medical center’s institutional review board. The original prospective observational study for which the dataset was obtained was conducted in the Pediatric Intensive Care Unit (PICU), Progressive Care Unit (PCU, a 25-bed step-down unit), Cardiac Intensive Care Unit (CICU), Operating Suites, and catheterization laboratory at a large academic children’s hospital in the United States. The dataset consisted of a convenience sample of 104 patients. Enrollment included children age 1–12 years between January 2018 and December 2018 (Table 1 and Supplementary Figure 1). An independent sample of adult patients (*n* = 30, mean age = 59 ± 20 years) was used as a validation cohort with clinician and device CRT collected in a similar fashion (Shinozaki et al., 2019b).

**TABLE 1:** Demographic and clinical characteristics of study and validation cohort.

Study cohort characteristics	Patients (*n* = 99)
Age, year, mean (SD), range	6.1 (3.9), 1–12
Weight, kg, median (SD), interquartile range	18.2 (20.9), 13.4–31.7
Sex, n (%)	
Male	58 (59)
Female	41 (41)
Study location, n (%)	
Operating room	56 (56)
Pediatric intensive care unit	26 (26)
Cardiac intensive care unit	12 (12)
Progressive care unit	4 (4)
Catheterization lab	1 (1)
Digit probe applied to (other digit visually assessed), n (%)	
Second digit	51 (51)
Third digit	48 (48)
First digit compressed, n (%)	
Second digit, Pointer	47 (47)
Third digit, Middle finger	52 (52)
Category of clinical diagnosis, n (%)	Surgical patients, 67*
	Head and neck surgery, 24
	Abdominal surgery, 7
	Cardiac surgery, 7
	Miscellaneous, minor procedure, 7
	Orthopedic surgery, 6
	Ophthalmologic surgery, 5
	Craniofacial surgery, 4
	Dental surgery, 4
	Urologic surgery, 2
	Neurosurgery, 1
	Acute respiratory failure, 15
	Acute cardiorespiratory failure, 10
	Chronic respiratory failure, 3
	Septic shock, 3
	Acute neurologic injury, 1
Individual curve CRT, clinician-determined	
Flash, n (%)	134 (28)
Normal, n (%)	269 (55)
Prolonged, n (%)	82 (17)

*Surgical patients includes post-operative patients in the ICU.

**FIGURE 1:**
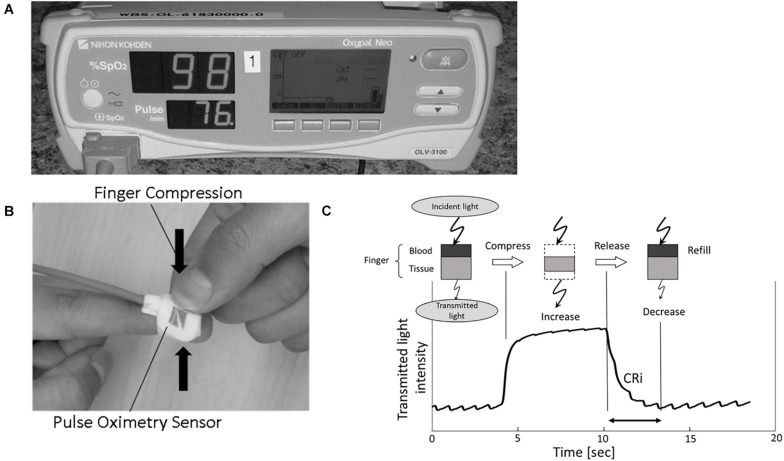
**(A,B)** Images display the modified pulse oximeter device and finger sensor. **(C)** Schematic showing device functioning. Incident light is transmitted through the patient fingertip. During fingertip compression, blood exits the fingertip and TLI increases. TLI falls as blood returns to the digit during capillary refill. CRi, Capillary Refill index; TLI, transmitted light intensity.

Capillary refill curves were obtained by a device using an age appropriate oxygen saturation (SpO2) sensor (TL-272 for larger children and TL-274 for smaller children; Nihon Kohden, Tokyo, Japan) connected to a pulse oximeter (OLV-3100; Nihon Kohden) (Figures 1A,B). A light emitting diode placed on the patient’s finger emits red and infrared light from the nail bed through the fingertip where a sensor detects the quantity of transmitted light, called the transmitted light intensity (TLI). TLI is equal to the difference between the light emitted and light absorbed by finger tissue and blood. The difference in TLI during a compression and release is proportional to the “thickness” (or volume) of blood present in the fingertip (Figure 1C; Oi et al., 2018). After pressure is applied and then released by a clinician, a descending TLI curve is generated. Capillary Refill index, CRi, is calculated as the time (seconds) between the compression release and return to 90% baseline in TLI. The TLI waveform is available on the right screen of pulse oximeter OLV-3100 during the CRi measurement process and CRi is calculated and presented on the screen upon completion of capillary refill measurement.

A combination of board certified pediatric intensivists, anesthesiologists, and experienced respiratory therapists who clinically perform CRT on a regular basis performed and measured the CRT for each patient. The device was randomly placed on each patient’s second or third digit. The clinician compressed either the second and third digit for 5 s. Following the pressure release, CRT was measured by the device or clinician depending on which finger was compressed. For the non-device finger, clinicians verbalized when full capillary refill had occurred; this time was recorded by study personnel with a chronograph. For the device finger, the TLI before, during, and after finger compression was recorded at 0.016 s intervals, creating a capillary refill curve (Figure 1C). Alternate finger compression was repeated five times and generated five paired CRT measurements for each patient. Device measurements were taken with at least 1 min in between finger compressions, with total time less than 15 min per patient for collection of 10 data points.

### Supervised Machine Learning Model Selection

Supervised machine learning is a learning paradigm in which a model is trained to map an input domain to an output range based on a previously observed set of input-output pairs, or training data (Russell and Norvig, 2010). Using clinician judgment as the *reference standard*, we trained three machine learning models to classify inputs as either flash CRT or not using gradient boosting (XGBoost), support vector machine (SVM) with radial basis function kernel, and logistic regression techniques. We performed the same analyses for prolonged CRT. Separate flash and prolonged CRT models were created to enable feature importance analysis relative to the type of CRT (vasodilated vs. vasoconstricted). Prior study of pulse oximeter waveform features in vasoconstricted and vasodilated states is sparse; useful information can be gained by comparing which features are important in algorithm performance in these different states. The use of three machine learning techniques allowed for comparison of performance. Different machine learning classifier models have varying capacity relative to their ability to learn different geometries (i.e., linear vs. non-linear) for decision boundaries. At the same time, a model with excess capacity given the available data and model input features may be easily overfit to the training data. Therefore, we elected to consider a linear model (logistic regression) and two non-linear models (XGBoost and SVM) to compare performance over a range of model capacities. We chose XGBoost and SVM as our non-linear models because they have performed well on many recent biomedical research studies and typically have fewer learning parameters than more complex models (e.g., deep-learning) which reduces the risk of overfitting (Mani et al., 2014; Masino et al., 2019a; Pang et al., 2019; Zabihi et al., 2019).

### Feature Selection and Model Training

Model input included statistical features extracted from time series data using the Python *tsfresh module* (Christ, 2019). All six models were trained using the same set of 10 features. The application of machine learning to pulse oximeter waveform analysis in CRT prediction is poorly studied, and as such, we primarily selected features that intuitively correlate with a graphical representation of blood return to finger capillary beds. These seven features were: maximum slope, standard deviation, mean, kurtosis, time of first minimum, skew, and area under the curve (AUC). Three additional features, ΔAb (before and after finger compression), and time series complexity were also included based on prior literature and proposed physiologic mechanism (Table 4).

All models were trained using nested cross validation (CV) which enables validation with all samples and model hyperparameter optimization (Supplementary Figure 2; Mani et al., 2014; Masino et al., 2019b). For the flash CRT dataset, training occurred by randomly dividing the data into 10-folds. Training and evaluation then proceeded in an iterative manner over the 10-folds. For each iteration, 1-fold is held out for validation, while the remaining folds are used for training and hyperparameter selection. This procedure yields 10 performance estimates and 10 hyperparameter selections (i.e., one for each fold). The same process was repeated for the prolonged CRT dataset but with 5 iterations instead of 10 based on a smaller number of prolonged samples. For the final model, the median value for selected hyperparameters over the folds was utilized. The *Python scikit-learn* and *Python XGB* libraries were used for all training and analysis (Pedregosa et al., 2011; Chen and Guestrin, 2016; XGBoost, 2020).

### Statistical Analysis

Patient characteristics were summarized by frequencies and proportions for categorical variables, and means and standard deviation along with ranges for continuous variables. For all models and non-machine learning CRi, the performance was assessed using receiver operating curve (ROC) analysis, with ROC reported as an average over the values obtained for each validation fold (five for prolonged models, 10 for flash models) of the nested cross validation procedure described above. Friedman Rank Sum test was implemented to assess whether ROC curves were different among the three classifiers. *Post-hoc* pairwise testing was applied to determine differences between individual classifiers. Precision-recall curves were also generated. Precision, or positive predictive value, is the ratio of true positives divided by the sum of true positives and false positives, and is the ability of the classifier to not label a negative (not flash or not prolonged) waveform as positive (flash or prolonged). Recall, or sensitivity, is the ratio of true positives divided by the sum of true positives and false negatives, and is intuitively the ability of the classifier to appropriately identify all positive samples (sensitivity). Additionally, the best performing prolonged CRT model was evaluated with an independent sample of adult patients with clinician and device data collected in a similar fashion (*N* = 90 measurement pairs) (Shinozaki et al., 2019b). This cohort consisted of 32% (*n* = 29) of waveforms with prolonged CRT by clinician assessment; there were no samples judged as flash CRT by clinicians and as such the flash CRT model could not be applied.

Permutation feature analysis utilizing the Python *ELI5* library was performed on the model with greatest mean AUC to determine the relative importance of each feature (Pedregosa et al., 2011; Korobov, 2016). In this technique, each feature is permuted, i.e., the subject values for that feature are shuffled randomly across samples such that the feature no longer provides useful information to the model. The degree that model performance decreases is indicative of feature importance to the model (Pedregosa et al., 2011).

We also ran two tests to identify sources of possible bias in our data collection, given that each patient provided five measurement pairs, each of which was treated as independent in our model training. We calculated the intraclass correlation coefficient (ICC) across all clinician-generated CRT values and all machine-generated CRi values to identify level of agreement among measurements. We also ran an analysis of variance (ANOVA) to assess whether the order of data acquisition (ordinal number 1–5) correlated with CRT or CRi values.

## Results

### Patients

Ninety-nine patients, age 1–12 years, and 485 pulse oximeter waveforms were included in algorithm training (Table 1).

### Model Development and Internal Validation

**FIGURE 2:**
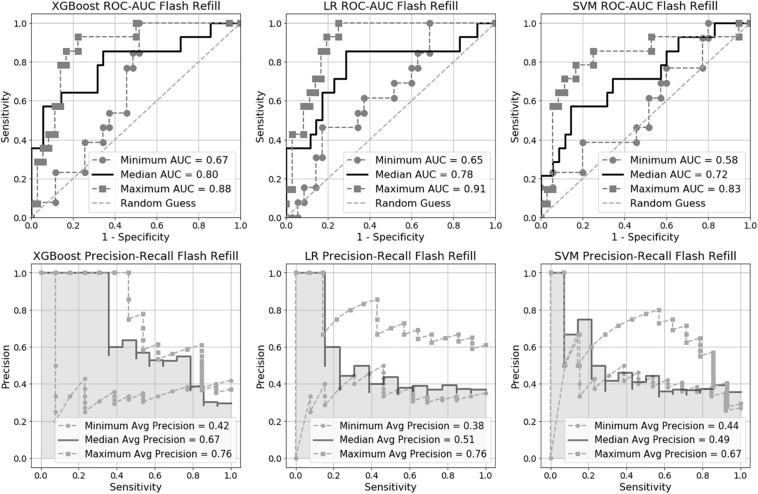
Receiver Operating Characteristic Area Under the Curve (ROC-AUC) and precision-recall for flash capillary refill time (CRT) models.

**FIGURE 3:**
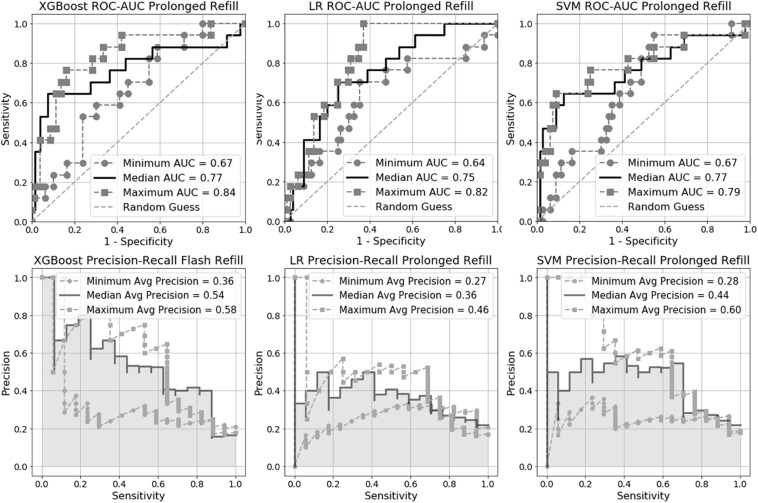
Receiver Operating Characteristic Area Under the Curve (ROC-AUC) and precision-recall for prolonged capillary refill time (CRT) models.

We trained six machine learning models in total, three for flash CRT detection and three for prolonged CRT detection. The AUC performance and precision-recall for each flash and prolonged model are presented in Figures 2, 3, and Table 2. For flash CRT detection, XGBoost had a greater numerical mean AUC and mean precision than logistic regression and SVM models. XGBoost had a sensitivity of 0.42 (95% CI 0.42–0.43), and specificity of 0.80 (95% CI 0.78–0.82). All ML models outperformed the non-ML reference standard, CRi, defined by the time that the TLI returns to 90% of its original baseline value. For prolonged CRT detection, XGBoost also had a numerically greater mean AUC and mean precision than logistic regression and SVM models. XGBoost for prolonged CRT detection had a sensitivity of 0.31 (95% CI 0.25–0.37), and specificity of 0.87 (95% CI 0.86–0.89). For the XGBoost model, optimal hyperparameters are reported in Supplementary Table 1, additional performance metrics in Supplementary Table 2, and learning curve analysis in Supplementary Figure 3.

**TABLE 2:** Mean Area Under the Curve and precision for each machine learning algorithm and capillary refill index.

	Flash CRT		Prolonged CRT	
		
Algorithm	AUC, Mean (95% CI)	Precision, Mean (95% CI)	AUC, Mean (95% CI)	Precision Mean (95% CI)
XGBoost	0.79 (0.75–0.83)	0.63 (0.56–0.69)	0.77 (0.72–0.82)	0.5 (0.46–0.54)
Logistic regression	0.77 (0.71–0.82)	0.56 (0.49–0.63)	0.73 (0.68–0.78)	0.37 (0.30–0.43)
Support vector machine	0.72 (0.67–0.76)	0.53 (0.47–0.58)	0.75 (0.70–0.79)	0.43 (0.32–0.54)
CRi	0.67 (0.63–0.71)	Not calculated	0.72 (0.68–0.76)	Not calculated

**CRT, Capillary Refill Time; AUC, Area Under the Curve; CRi, Capillary Refill index; CI, Confidence Interval.**

Based on the Friedman Rank Sum test, the null hypothesis that all three flash capillary refill models (Logistic Regression, SVM, XGBoost) have area under the ROC curve values (from the 10 CV folds) from the same distribution is rejected with a *p*-value of 0.007 suggesting there is a statistically significant difference among the group of models. *Post-hoc* pairwise testing using the Wilcoxon signed-rank test indicates a significant difference (*p*-value 0.009) between XGBoost and SVM. All other pairs were not statistically significant. Applying the same procedure to the prolonged capillary refill model failed to reject the null hypothesis, suggesting the models have equivalent performance.

### External Validation With Adult Dataset

When applied to an independent sample of adults (30 patients with 90 pairs of CRT measurements), the prolonged SVM showed good agreement with clinician-judged CRT, with an AUC of 0.88 and precision of 0.79 (Supplementary Figure 4). In comparison, the non-machine learning CRi had less agreement with clinician-determination, with an AUC of 0.84 when applied to the same cohort (Morimura et al., 2015; Oi et al., 2018).

### Feature Importance Analysis

For the XGBoost flash CRT model, ΔAb post-compression, time complexity, and kurtosis were the most influential variables in the model. For the XGBoost prolonged CRT model, time complexity, point of first minimum, and ΔAb pre-compression were the most influential variables (Table 3). The feature explanation and proposed physiological significance of these variables are shown in Table 4.

**TABLE 3:** Feature importance analysis for XGBoost flash and prolonged capillary refill time models.

XGBoost feature importance				

Importance rank	Flash model		Prolonged model	
		
	Feature	Weight	Feature	Weight
1	ΔAb post-compression	0.12	Time complexity	0.06
2	Time complexity	0.11	Point of first min.	0.05
3	Kurtosis	0.05	ΔAb pre-compression	0.05
4	Area Under the Curve	0.04	Skew	0.04
5	ΔAb pre-compression	0.04	Maximum slope	0.03
6	Point of first min.	0.03	Area Under the Curve	0.03
7	Skew	0.02	Kurtosis	0.03
8	Standard deviation	0.02	Standard deviation	0.02
9	Mean	0.01	ΔAb post-compression	0.01
10	Maximum slope	0.01	Mean	0.01

**ΔAb: maximal difference between the infrared and red light detection by the device × hemoglobin density × tissue thickness.**

**TABLE 4:** Graphical and physiologic explanation of model features.

Feature name	Feature explanation	Proposed physiologic significance
Point of first minimum	The time point from release where the minimal value of the dataset occurs	Earlier point of first minimum indicates earlier return of blood to the capillary bed, correlating with faster capillary refill
Maximum slope	The absolute value of the maximum (negative) slope of the dataset	Greater maximal slope indicates greater filling speed of blood into the capillary bed, correlating with faster capillary refill
Kurtosis	The sharpness of the peak of a frequency distribution curve–greater kurtosis implies a steeper descent from point of release	Greater kurtosis indicates greater filling speed of blood into the capillary bed, correlating with prolonged capillary refill
Skew	The measure of asymmetry of the sample, samples with longer and thicker tails are more skewed	Greater skew indicates more gradual return of blood to the capillary bed, correlating with prolonged capillary refill
Mean	The average value of the dataset	A greater mean value indicates overall less blood and tissue in the capillary bed (greater TLI = less blood and tissue present), implication for capillary refill speed uncertain
Standard deviation	A calculated quantity indicating the extent of variation for the dataset	A greater standard deviation correlates with a greater difference between TLI at release and TLI at minimum, implication for capillary refill speed uncertain
ΔAb (pre- and post-compression)	The maximal difference between the infrared and red light detection by the device × hemoglobin density × tissue thickness	Oxygenated Hb absorbs more infrared light; deoxygenated Hb absorbs more red light. The greatest difference between these values could imply a reduced percentage of Hb saturation which may correlate with abnormal CRT (prolonged for flash). Anemia, peripheral vasoconstriction, shock, strong or sharp pain may also increase ΔAb, correlating with abnormal CRT. ΔAb has correlated with degree of lactic acidosis among adult emergency room patients in shock (Oi et al., 2018).
Time series complexity	Complexity of a time series is determined by the number and degree of peaks and valleys in the curve	Time series complexity may represent variable vasoconstriction of individual capillary beds within the digit postulated to be present with abnormal capillary refill

### Intraclass Correlation Coefficient (ICC) and Analysis of Variance (ANOVA)

The ICC for clinician-measured CRT was 0.89, indicating good to excellent agreement among measurements. The ICC for machine-measured CRi was 0.39, indicating poor agreement. ANOVA testing indicated that there was no association between order of measurement and length of clinician measured CRT (*p* = 0.44–0.60), but that there was a significant linear association between order of measurement and machine-measured CRi (*p* = 0.005). There was a negative linear trend shown in Supplementary Figure 5, meaning that subsequent CRi measurements were shorter based on order of measurement. To estimate the degree of effect, we plotted CRi values (y axis, seconds) against ordinal numbers indicated position of measurement (x axis, 1–5), and calculated a slope of −0.176, *p* = 0.0065.

## Discussion

Our results represent the first application of supervised machine learning to pulse oximeter waveforms analyzing capillary refill time. We utilized gradient boosting (XGBoost), SVM with radial basis function kernel, and logistic regression. XGBoost had the highest mean AUC with good internal validation. We found the machine learning based models had higher mean AUC results when compared to the non-machine learning calculation CRi for flash and prolonged models with internal validation. Notably, the XGBoost algorithm for prolonged CRT also showed good performance to detect prolonged CRT in the external validation cohort with adult patients (mean AUC 0.88).

Feature importance analysis showed several interesting findings (Table 3). For both flash and prolonged CRT SVMs, time complexity and ΔAb were among the top three most influential variables. Given that time complexity represents the randomness (degree of peaks and valleys of the waveform), it is postulated that this may represent heterogeneous vasoconstriction of individual capillary beds within the digit when very fast or prolonged capillary refill is present. With increased heterogeneity, more peaks and valleys will be present in the waveform itself. ΔAb may correlate with degree of hypoxia, anemia, or amount of blood in the fingertip, and has correlated with degree of lactic acidosis in a prior publication (Oi et al., 2018). Given that one explanation for ΔAb is decreased blood in the finger, there was concern that this measure simply correlated with the pressure the clinician applied during measurement of CRT, introducing bias into the model. There was weak correlation between ΔAb-pre compression or post compression and the force applied by clinicians (*r* = 0.15, *r* = 0.16, respectively). As such, it appears unlikely that ΔAb simply represents the degree of clinician force applied and instead has a physiologic explanation (Table 4).

Although the novel waveform analysis of the device is early stage, the data shows good agreement with clinician-judged flash or prolonged CRT even with only 10 waveform variables included. Our machine learning model performance may further improve with the addition of clinical variables. A recent publication showed that CRi in adults were significantly associated with age, serum blood urea nitrogen, serum creatinine, fingertip temperature, red blood cell count, and albumin (Shinozaki et al., 2019a). As such, including these variables or other parameters, such as the presence of vasoactive infusions, diagnosis, or vital sign data, may yield even better model performance. Automated pressure application to generate finger blanching and automate capillary refill generation utilizing an insufflation cuff is currently being developed. We hope that with this automated CRT waveform generation and further refinement of CRi estimation that the device will eventually allow for automated, recurring, and reliable peripheral perfusion assessment to guide clinical care.

Is clinician reported CRT reliable and the correct comparator for a machine learning algorithm comparison? Inter-rater reliability reporting has been somewhat variable. Studies assessing visually assessed CRT in the emergency department have reported moderate to good kappa values, κ range 0.30–0.54 (Gorelick et al., 1993; Alsma et al., 2017). However, other reports indicate high capillary refill reliability. van Genderen et al. (2014) reported good to excellent inter-observer reliability between two examiners showing κ = 0.91 (95%CI 0.80–0.97) and 0.74 (0.52–0.89) from different postoperative days. Ait-Oufella, similarly, reported excellent inter-rater concordance calculated at 80 and 94% for finger and knee CRT measurements. The latter two studies utilized strict standardized CRT protocols and chronographs. In line with these findings, Fleming’s systematic review suggested that explicit protocolization of CRT measurement and use of chronographs may improve inter-rater reliability (Fleming et al., 2015b).

Clinical utility of machine learning based CRT measurement may be promising. A recently published trial randomized adult patients receiving septic shock treatment into two groups: one group aimed at normalizing peripheral perfusion using CRT and second group normalizing lactate levels. Their results suggested CRT guided sepsis treatment is feasible, reporting a 28-day all-cause mortality hazard ratio of 0.72 (95% CI 0.55–1.02; *P* = 0.06) in the peripheral perfusion group with significantly improved Sequential Organ Failure Assessment score at 72 h compared to the lactate group (Hernández et al., 2019). Future pediatric studies need to address the feasibility and potential benefit of CRT guided shock treatment over the conventional clinical indicators of current use. In light of this data, the ability to reliably and automatically detect capillary refill (or a perfusion surrogate, CRi) could have significant implications for management of critically ill patients in shock.

### Study Limitations and Sources of Model Bias

Several factors which were not controlled in this study might have introduced biases. These factors include the amount of pressure applied by clinicians during finger blanching, ambient temperature, right or left hand selection, and patient position in bed. The ICCs calculated for our CRT and CRi measurements were quite different (0.89 vs. 0.39), reflecting a potential anchoring bias in physician measured CRT: a tendency to generate similar values in repeated measurements. While the repeated measurements within each subject may bias the overall ML model, lower ICC in CRi measurement indicates a relatively large variance in the waveform parameters, which may reduce this bias from the repeated measures. The order of the measurement within the subject was associated with the length of CRi. This may be due to the measurement noise and measurement position within each subject, though it may also represent the true physiologic state as inter-measurement intervals was short, approximately 1 min.

Our study did not include an external validation set for the XGBoost flash CRT model because no adult patients in this dataset had flash CRT. Therefore our model performance relied on internal cross-validation only. The study did include an external validation set for prolonged CRT, however the external validation was for an adult patient population which may have different capillary refill time characteristics than children. Our sample size did not allow us to externally validate the model on the same pediatric or adult population. This will be an important research topic in the future. The dataset utilized was a convenience sample of primarily peri- and intra-operative patients requiring intensive care unit admission, a minority of whom had evidence of septic shock, from a single large academic children’s hospital and thus results may not be generalizable to a more broad pediatric population. We suggest further study in patients with more severe critical illness. Each patient generated five paired clinician-waveform measurements; as such some of the model inputs may not be truly independent. We defined prolonged CRT as >2.0 s which is more stringent than the another commonly accepted definition of prolonged CRT as >3.0 s (Fleming et al., 2015b; American Heart Association, 2016; Davis et al., 2017). We suggest future study assessing algorithm ability to identify CRT >3.0 s.

## Conclusion

Our study showed the first successful application of supervised machine learning techniques to analyze pulse oximeter waveforms to detect flash and prolonged capillary refill. Utilizing clinician-judged CRT as the reference standard, we trained six separate models that showed good internal validation in detection of both flash and prolonged CRT. Gradient boosting (XGBoost) also showed good external validity for the prolonged CRT detection algorithm. ΔAb and time complexity were revealed as novel features important in both flash and prolonged CRT detection. These results suggest the feasibility ML application to pulse oximeter waveforms in characterizing peripheral perfusion even with a small testing cohort. Further study of waveform analysis with other clinical and laboratory measures of microcirculation is needed.

## Data Availability Statement

The raw data supporting the conclusions of this article will be made available by the authors, without undue reservation, to any qualified researcher. Requests to access the datasets should be directed to RH, hunterrb@email.chop.edu.

## Ethics Statement

The studies involving human participants were reviewed and approved by the Children’s Hospital of Philadelphia Institutional Review Board. Written informed consent to participate in this study was provided by the participants’ legal guardian/next of kin.

## Author Contributions

RH primary author, lead development of idea, formulation of graphical features, and primary drafting of manuscript. SJ helped with technical implementation and execution of algorithm, intimately involved in development of algorithm features and analysis, expert in device functionality, and helped collect primary data. AN and VN primary clinical mentors, they are overseeing experimentation of the capillary refill device in pediatrics, helped guide project development, and heavily influenced manuscript. AJN and NN involved in primary data collection and reviewed abstract and manuscript drafts. KSh, TL, KSa, and LB obtained validation dataset, helped edit multiple drafts of manuscript, and served as clinical and technical advisors during drafting process. AM lead author, oversaw the whole project with emphasis on algorithm development, and checked for technical feasibility and sound process. All authors contributed to the article and approved the submitted version.

## Conflict of Interest

SJ and KSa were employees of Nihon Kohden Innovation Center, Cambridge, MA, United States. NN had research/consulting relationships with Draeger Medical, Smiths Medical, Philips/Respironics, Aerogen, Actuated Medical, and VERO-Biotech. LB was a compensated member of the Scientific Advisory Board of Nihon Kohden Corporation. Both Children’s Hospital of Philadelphia and Nihon Kohden Corporation hold intellectual property of capillary refillometer technology. The remaining authors declare that the research was conducted in the absence of any commercial or financial relationships that could be construed as a potential conflict of interest.
