# Supplementary material for: Supervised Machine Learning Applied to Automate Flash and Prolonged Capillary Refill Detection by Pulse Oximetry
Source: Front Physiol. 2020 Oct 6;11:564589. doi: 10.3389/fphys.2020.564589 (PMC7574820; doi:10.3389/fphys.2020.564589)
Supplement: Supplementary file 1 [file Data_Sheet_1.docx]

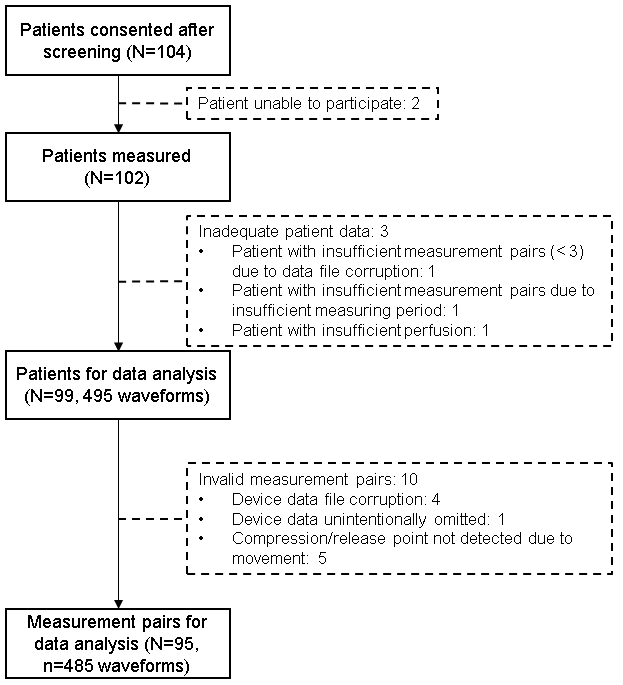


**Supplementary Figure 1.** Study flow diagram.


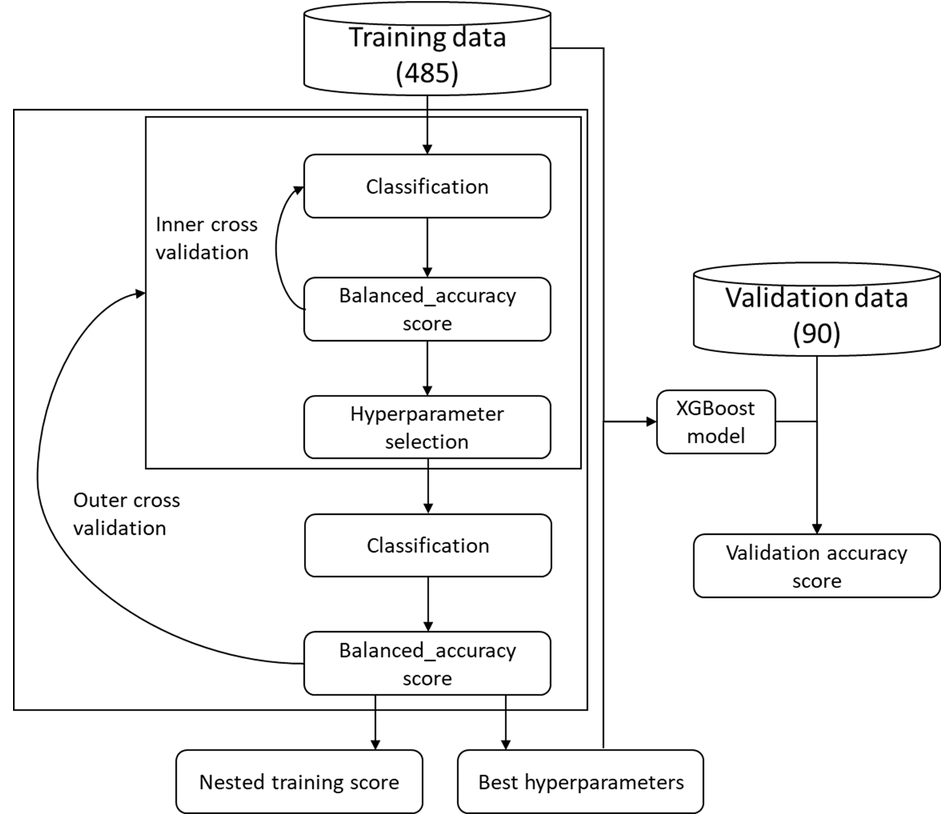


**Supplementary Figure 2.** Flowchart of the supervised machine learning algorithm training.


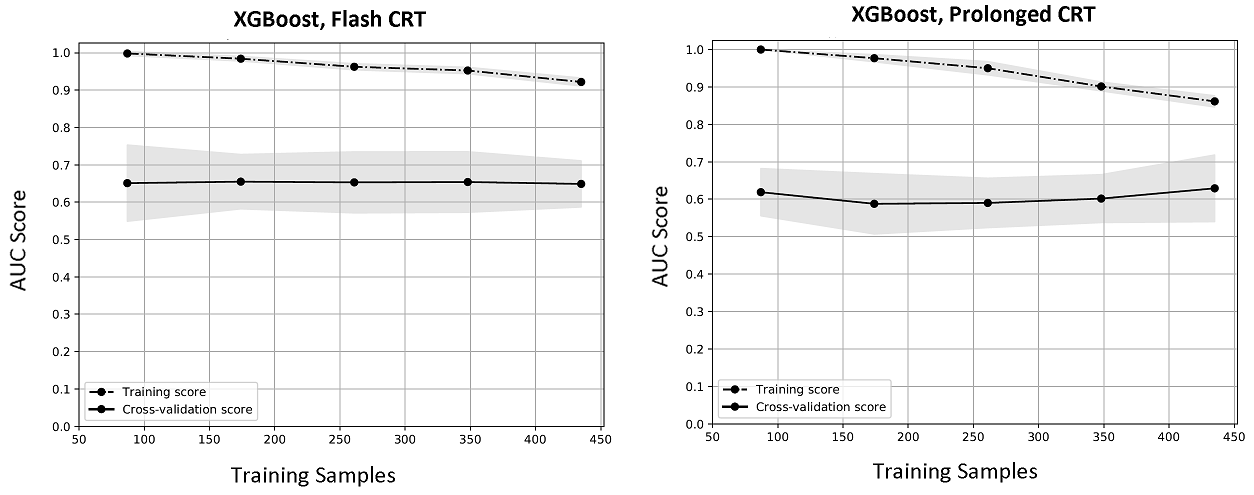


**Supplementary Figure 3.** Learning curves for XGBoost flash CRT and prolonged CRT detection models. Curves show a slight downward trend in the training scores for the learning curves of the XGBoost flash CRT model in their asymptotic limits, suggesting that the model has insufficient capacity to distinguish all possible CRT waveforms. Using clinician judgment of CRT as the “reference standard” likely introduces bias caused by noise in the training data. There was slight downward trend of the training curve at ~250 samples and very slight upward trend in the cross validation curve for the prolonged model. This may indicate a model bias, or systematically prejudiced results which are overfit to the training data. If this is the case, additional waveform and clinical features would likely improve model performance. The cross-validation score did not achieve the same score as the training-score when all training samples were used for either flash or prolonged CRT, though they did trend toward the same asymptote (between 0.7-0.8) (final difference >20%). This also likely indicates that some over-fitting is present in the model. Despite these analyses, the model had excellent performance when validated in the external dataset (mean AUC 0.88), suggesting minimal impact of overfitting.


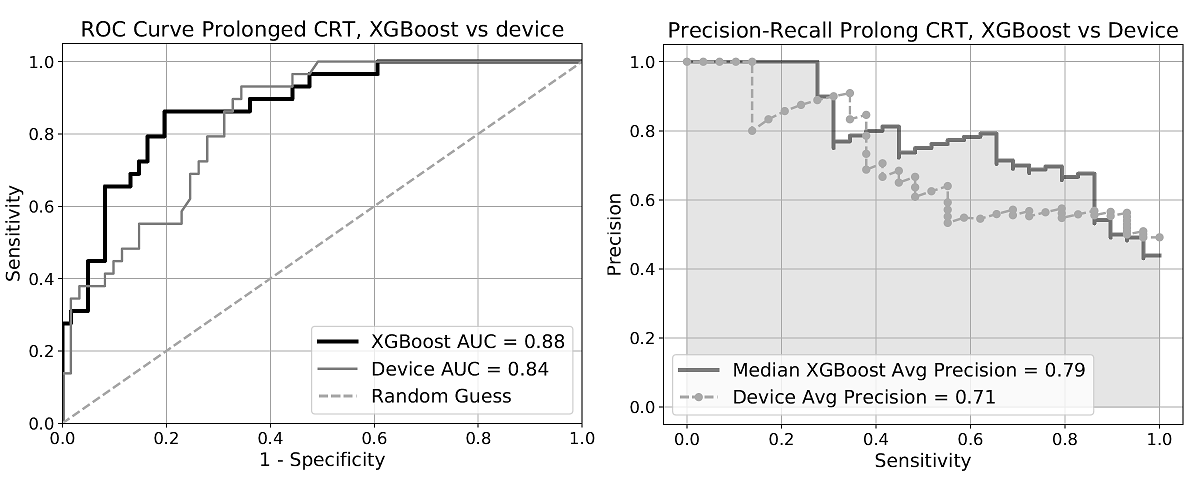


**Supplementary Figure 4.** Receiver Operating Characteristic-Area Under the Curve (ROC-AUC) and precision-recall curve for prolonged CRT model compared to non-machine learning model applied to an external cohort of adult-derived CRT pairs**.**

**
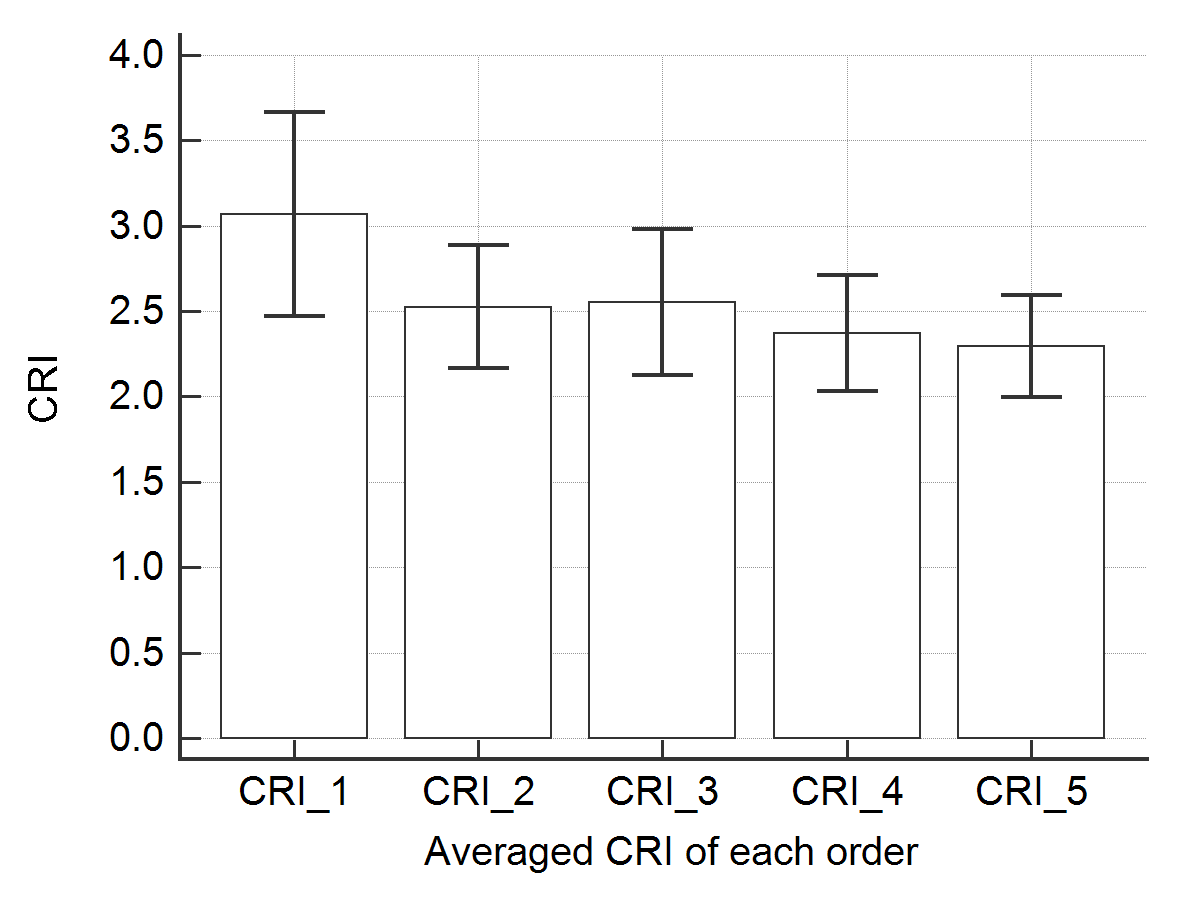
**

**Supplementary Figure 5.** Averaged Capillary Refill Index (CRi), grouped by order of measurement (first measurement through last) displays a mild negative trend between CRi and order of measurement.

|  | XGBoost Optimal Hyperparameters, value (n/total iterations) | |
| --- | --- | --- |
| Hyperparameter | **Flash Model** | **Prolonged Model** |
| Max Depth | 5 (4/10) 4 (3/10), 3 (3/10) | 5 (3/5), 4 (2/5) |
| Estimators | 25 (6/10), 100 (3/10), 50 (1/10) | 25 (2/5), 100 (2/5), 50 (1/5) |
| Gamma | 1 (5/10), 0 (3/10), 0.1 (2/10) | 0 (2/5), 0.1 (3/5) |
| Min child weight | 0.1 (4/10), 0.5 (3/10), 1 (3/10) | 1 (3/5), 0.5 (1/5), 0.1 (1/5) |

**Supplementary Table 1**. Optimal hyperparameters for XGBoost model.

| XGBoost Performance Metric | Flash CRT; Mean (95% CI) | Prolonged CRT; Mean (95% CI) |
| --- | --- | --- |
| Accuracy | 0.75 (0.71-0.79) | 0.85 (0.84-0.86) |
| Positive Predictive Value | 0.57 (0.47-0.66) | 0.71 (0.63-0.78) |
| Negative Predictive Value | 0.63 (0.56-0.69) | 0.87 (0.86-0.88) |

**Supplementary Table 2.** Additional XGBoost performance metrics. CRT: Capillary Refill Time. CI: Confidence Interval.
